# Comparative Genomic Analysis Shows That Avian Pathogenic *Escherichia coli* Isolate IMT5155 (O2:K1:H5; ST Complex 95, ST140) Shares Close Relationship with ST95 APEC O1:K1 and Human ExPEC O18:K1 Strains

**DOI:** 10.1371/journal.pone.0112048

**Published:** 2014-11-14

**Authors:** Xiangkai Zhu Ge, Jingwei Jiang, Zihao Pan, Lin Hu, Shaohui Wang, Haojin Wang, Frederick C. Leung, Jianjun Dai, Hongjie Fan

**Affiliations:** 1 College of Veterinary Medicine, Nanjing Agricultural University, Nanjing, China; 2 Bioinformatics Center, Nanjing Agricultural University, Nanjing, China; 3 School of Biological Sciences, University of Hong Kong, Hong Kong SAR, China; 4 Shanghai Veterinary Research Institute, Chinese Academy of Agricultural Sciences, Shanghai, China; University of Helsinki, Finland

## Abstract

Avian pathogenic *E. coli* and human extraintestinal pathogenic *E. coli* serotypes O1, O2 and O18 strains isolated from different hosts are generally located in phylogroup B2 and ST complex 95, and they share similar genetic characteristics and pathogenicity, with no or minimal host specificity. They are popular objects for the study of ExPEC genetic characteristics and pathogenesis in recent years. Here, we investigated the evolution and genetic blueprint of APEC pathotype by performing phylogenetic and comparative genome analysis of avian pathogenic *E. coli* strain IMT5155 (O2:K1:H5; ST complex 95, ST140) with other *E. coli* pathotypes. Phylogeny analyses indicated that IMT5155 has closest evolutionary relationship with APEC O1, IHE3034, and UTI89. Comparative genomic analysis showed that IMT5155 and APEC O1 shared significant genetic overlap/similarities with human ExPEC dominant O18:K1 strains (IHE3034 and UTI89). Furthermore, the unique PAI I_5155_ (GI-12) was identified and found to be conserved in APEC O2 serotype isolates. GI-7 and GI-16 encoding two typical T6SSs in IMT5155 might be useful markers for the identification of ExPEC dominant serotypes (O1, O2, and O18) strains. IMT5155 contained a ColV plasmid p1ColV_5155_, which defined the APEC pathotype. The distribution analysis of 10 sequenced ExPEC pan-genome virulence factors among 47 sequenced *E. coli* strains provided meaningful information for B2 APEC/ExPEC-specific virulence factors, including several adhesins, invasins, toxins, iron acquisition systems, and so on. The pathogenicity tests of IMT5155 and other APEC O1:K1 and O2:K1 serotypes strains (isolated in China) through four animal models showed that they were highly virulent for avian colisepticemia and able to cause septicemia and meningitis in neonatal rats, suggesting zoonotic potential of these APEC O1:K1 and O2:K1 isolates.

## Introduction

*Escherichia coli* generally colonizes the mammalian intestinal tract commensally, but highly adapted *E. coli* clones can become true pathogens called “pathotypes”, some of which cause various lethal diseases after acquisition of specific virulent factors [1], [2]. These *E. coli* pathotypes can be broadly classified as intestinal pathogenic *E. coli* or extraintestinal pathogenic *E. coli* (ExPEC) based on the pathogenic types [3]. Intestinal pathogenic *E. coli* strains (IPEC) cause infection in the gastrointestinal system, while ExPEC strains cause urinary tract infections, newborn meningitis, abdominal sepsis, and septicemia in the extraintestinal system [2], [4]. ExPEC pathotypes are classically divided into four groups, based on the disease pathology, namely avian pathogenic *E. coli* (APEC), uropathogenic *E. coli* (UPEC), neonatal meningitis *E. coli* (NMEC), and septicemic *E. coli* [5]–[7].

In order to discriminate ExPEC from commensal and intestinal pathogenic *E. coli*, several molecular epidemiology approaches are used for ExPEC typing. The classical typing method is the identification of *E. coli* (O: K: H) serotypes, and highly virulent ExPEC isolates can be classified as several specific and predominant O1, O2 and O18 serotypes strains, which can express K1 capsule and are popularly isolated from human and avian colibacillosis [6], [8]–[10]. Related to above mentioned three O serotypes, O6 serotype strains are also highly virulent and popular among UPEC isolates [6], [11], and APEC O78 serotype strains are also frequently isolated from avian colibacillosis [6], [12]. The phylogroup typing method based on multilocus enzyme electrophoresis (MLEE) and several relevant DNA markers are generally used for identification of *E. coli* genetic and evolutionary characteristics. *E. coli* can be classified as four major phylogroups (A, B1, D and B2) in accordance with the studies of Clermont et al. [13]–[16], and an additional fifth group (E) [17]–[19]. Most ExPEC isolates belong to the mainly phylogroup B2 and a lesser group D, especially highly virulent ExPEC strains, while intestinal pathogens and commensals *E. coli* mainly belong to group A and B1 [20]. In addition, the phylogroup E contains almost all serotype O157:H7 strains [18], [19], [21]. Multilocus sequence typing (MLST) is currently most powerful typing system for the discrimination of bacterial population genetics [22]. The molecular epidemiology shows that phylogenetic diversity of *E. coli* isolates are unambiguously differentiated based on *E. coli* MLST data (clonal complexes and sequence types data) [17], [23]. ExPEC and IPEC isolates are generally distributed in distinct clonal complexes i.e. sequence type complexes, containing numerous sequence types (ST) for *E. coli* MLST database. The majority of ExPEC isolates are located in several specific ST complexes (95, 73, 131, 127, 141, et al.), which are called ExPEC dominated clonal complexes[24]–[27]. Phylogroup B2 ExPEC strians of serotypes O1, O2 and O18 are generally located in ST complex 95, and ExPEC isolates of ST complex 95 are popular objects for ExPEC genetic characteristics and pathogenesis in recent years [5], [6], [19], [27]–[29].

After its entry via inhalation of fecal dust, APEC colonizes at the avian respiratory tract, and causes local infections and then spreads to various internal organs, resulting in systemic infection in poultry. These APEC-associated systemic infections have been proven economically devastating to global poultry industries [6], [29]–[31]. The phylogroup B2 APEC strains isolated from avian colibacillosis mainly belong to O1:K1, O2:K1, and another O78 serotypes [6], [9]. The complete genomic sequence of APEC O1 (an O1:K1:H7 strain; ST95) is first determined, which shares high similarities with the genomes of human UPEC isolates [5]. APEC and NMEC ST95 serotype O18 isolates can both cause meningitis in the rat model and disease in poultry, suggesting that they might have no or minimal host specificity [32]. APEC O78 strain χ7122 (ST23) is the second genome that has been sequenced in APEC isolates, which keeps close relationship with human ST23 ETEC than that of APEC O1 and human ExPEC strains. APEC wild-type strain IMT5155 (O2:K1:H5; ST complex 95, ST140; B2 phylogroup) is often used as a classic infection strain of APEC pathogenicity to identify APEC virulence factors [33]–[35]. Due to close relationship of ExPEC O2:K1 serotype strains with extraintestinal infection between humans and animals, we reported the complete genome sequence of IMT5155 in order to unravel the evolutionary and genomic features of APEC O2 isolates. We further compared IMT5155 genome with other *E. coli* strains to identify APEC/ExPEC genetic characteristics. In addition, virulence and zoonotic potentials of APEC O1:K1 and O2:K1 serotypes isolates were assessed through animal models for pathogenicity testing.

## Materials and Methods

### APEC strain and the total DNA extraction

The avian pathogenic *E. coli* strain IMT5155 was isolated from a chicken with the typical clinical symptoms of avian colibacillosis at a German chicken farm in the year 2000 and were provided by Lothar H Wieler and Christa Ewers [33]. The IMT5155 cells were cultured in LB media to its exponential growth phase and harvested by centrifuge. The bacteria genomic DNA extraction was extracted using the Bacterial DNA Kit (Omega Bio-Tek, America).

### 454 pyrosequencing of the IMT5155 genome and assembly

A whole genome shotgun library was produced with 5 µg of the genomic DNA of IMT5155. The shotgun sequencing procedure followed the instruction of 454 GS Junior General Library Preparation Kit (Roche). In addition, an 8 kb insert paired end library was produced with 15 µg of the genomic DNA of IMT5155. The paired end sequencing procedure followed the instruction of 454 GS Junior Paired-end Library Preparation Kit (Roche). Paired-end reads were used to orientate the contigs into scaffolds. The DNA libraries were amplified by emPCR and sequenced by FLX Titanium sequencing chemistry (Roche). Two shotgun runs and one paired-end runs were performed based on their individual library. After sequencing, the raw data were assembled by Newbler 2.7 (Roche) with default parameters. Primer pairs were designed along the sequences flanking the gap regions for PCR gap filling. The complete sequences of IMT5155 chromosome and two plasmids have been deposited in GenBank (Accession numbers: CP005930, CP005931, and CP005932, respectively).

### Genome annotation of IMT5155

Glimmer 3.02 was used for gene prediction of IMT5155 complete genome [36]. The Glimmer results were corrected manually, and pseudogenes were investigated through genome submission check process for GenBank (http://www.ncbi.nlm.nih.gov/genomes/frameshifts/frameshifts.cgi), and small CDSs in intergenic regions were identified by IASPLS (Iteratively adaptive sparse partial least squares) [37]. Then, all the predicted ORF sequences were translated into protein sequences. BLASTp was applied to align all the above protein sequences against the NCBI non-redundant database (January, 2013) [38]. Protein sequences with alignment length over 90% of its own length and over 50% identity were chosen and the name of the best hit will be assigned to the corresponding predicted gene. rRNA operons were annotated by RNAmmer (http://www.cbs.dtu.dk/services/RNAmmer/), tRNA genes tRNAscan-SE Search Server (http://lowelab.ucsc.edu/tRNAscan-SE/), and tmRNA were annotated by tmRNA Database (http://rth.dk/resources/rnp/tmRDB/) with default parameters.

### Phylogenomic analysis of IMT5155 with other *E. coli* pathotypes

46 complete genomes and 1 draft genome of *E. coli* strains were downloaded from NCBI GenBank (File A in File S3). The othologous genes were identified by using the predicted genes of IMT5155 to align to all annotated genes of 47 *E. coli* by BLAT (the BLAST-like alignment tool) [39]. Those single copy IMT5155 genes over 90% of alignment length against all other *E. coli* strains were considered as the common genes, which composed the common genome of 47 *E. coli* strains. Then, all the common genes were aligned by MUSCLE and concatenated together [40]. Finally, the concatenated aligned genes were submitted to MrBayes with the GTR+G+I substitution model [41]. The chain length was set to 10,000,000 (1 sample/1000 generations). The first 2,000 samples were discarded as burn in after scrutinizing the trace files of two independent runs with Tracer v1.4 (http://tree.bio.ed.ac.uk/software/tracer/).

### Virulence genes and Genomic islands of IMT5155

The annotated genes were submitted to IslandViewer (http://www.pathogenomics.sfu.ca/islandviewer/genome_submit.php) and PAIDB (https://www.gem.re.kr/paidb/about_paidb.php) with default parameters for the identification of genomic islands s, i.e., pathogenecity island-like region [42], [43]. Then the annotated genes were submitted to VFDB database (http://www.mgc.ac.cn/VFs/) for the identification of virulence genes [38], [44]. Protein sequences with alignment length over 90% of its own length and over 50% identity were chosen from VFDB database, and the name of the best hit will be assigned to the corresponding predicted gene. Through online prediction and manual inspection, we obtained the detailed and precise information for IMT5155 GIs and virulence genes.

### Comparative genomic analysis

For comparative studies, common genes in chromosomes of other *E. coli* strains (APEC O1, CFT073, χ7122, MG1655, SE15, O157Sakai, IHE3034, CE10, 83972, NA114, UMN026, UTI89, E2348/69, RM12579, NRG857c, and UM146) shared with *E. coli* IMT5155 were identified and plotted along with all predicted genes in *E. coli* IMT5155 (with >90% alignment length and >50% identity). The similarities and differences of the predicted genes located in IMT5155 genomic islands were highlighted among the other *E. coli* strains.

p1ColV5155 and 5 plasmids (pAPEC-O2-ColV, pAPEC-O1-ColBM, pUTI89, pMAR2, and pO83-CoRR) were used for plasmid comparative analysis and synteny analysis. The common genes in 5 plasmids shared with p1ColV_5155_ were identified and plotted along with all predicted genes in p1ColV_5155_ as well as some functional genes. All genes of 5 plasmids were aligned with all genes predicted in p1ColV_5155_ respectively. Then, the aligned genes (with >90% alignment length and >50% identity) were shown for synteny analysis. The scripts for comparative ORF analysis and GIs distribution between IMT5155 and other *E. coli* strains were shown in File B in File S3.

### The distribution analysis of 10 sequenced B2 ExPEC pan-genome virulence genes among all sequenced *E. coli* strains

The homologous and non-orthologous genes in genomes of 10 sequenced B2 ExPEC strains (NA114, UTI89, IHE3034, IMT5155, APEC O1, S88, CFT073, Clone Di14, ABU83972, 536) were identified by this standard: homology genes, gene sequence identity ≥80% and coverage ≥80%, otherwise it was a non-orthologous gene. The total genes of the homologous and non-orthologous genes of those genomes represent the pan-genome of 10 sequenced B2 ExPEC genomes. The genes of pan-genome for 10 sequenced B2 ExPEC were translated into protein, and then protein of 10 sequenced B2 ExPEC pan-genome were submitted to VFDB database (with >90% alignment length and >50% identity) [38], [44]. Then all predicted virulence genes were one by one manually verified through a large number of references about ExPEC virulence factors, and the confirmed virulence-associated genes were classified as six categories: adhesins, invasins, toxins, iron acquisition/transport systems, polysialic acid synthesis, and other virulence genes. For distribution analysis of virulence genes, common genes in 46 *E. coli* genomes (selected consistent with phylogenomic analysis) (File A in File S3) shared with virulence genes of 10 sequenced B2 ExPEC pan-genome were identified with >90% alignment length and >50% identity, and highlighted among all 46 sequenced *E. coli* strains expect draft PCN033 genome sequence. The scripts for virulence genes statistics and heat-map for virulence gene distribution were shown in File B in File S3.

### Pathogenicity testing

All animal experimental protocols were approved by the Laboratory Animal Monitoring Committee of Jiangsu Province, China.

#### (i) Chicken embryo lethality assay (ELA)

The ELA model was performed to evaluate lethality in chicken embryos for IMT5155 and other APEC strains, as previously described [5], [32]. Briefly, approximately 500 CFU of each cultured bacterial were inoculated into the allantoic cavity of a 12-day-old, embryonated, specific-pathogen-free egg (Jinan SAIS Poultry Co. Ltd.), and 20 eggs were successively inoculated for every experimental group. PBS-inoculated and uninoculated were used as negative controls. The inoculated eggs were checked daily, and embryo deaths were recorded for 4 days.

#### (ii) Chick colisepticemia model

IMT5155 and other APEC strains to cause avian colibacillosis were assessed for chick lethality, as previously described [5], [32]. Briefly, group of 10 1-day-old SPF chicks (QYH Biotech) were inoculated intratracheally with 0.1 ml bacteria suspensions (approximately 10^7^ CFU) for APEC and other strains. The groups for chicks inoculated with PBS and MG1655 acted as negative controls. Measuring time for mortality were 7 days after postinfection. Deaths were recorded, and the survivors after 7 days were euthanatized, and all tested chicks in each group were dissected and examined for lesion scores (ranked from 0 to 3 in accordance with the presence of airsacculitis, pericarditis, and perihepatitis). The air sacs, blood in heart, and brain of all tested chicks were picked using inoculation loops, and then plates of MacConkey agar were crossed by inoculation loops and cultured at 37°C overnight.

#### (iii) Mouse sepsis model

The mouse sepsis model for virulence evaluation of ExPEC isolates was performed on the basis of previously described methods [28], [45], [46]. Approximately 10^7^ CFU (0.2 ml) of bacteria suspensions for APEC and other strains were injected intraperitoneally into 8-week-old imprinting control region (ICR) mice, and every group contained 10 mice. Mice for health status were observed twice daily during 3 days postinfection, which was score on a 5-step scale (1 = healthy, 2 = minimally ill, 3 = moderately ill, 4 = severely ill, 5 = dead) with the worst score as the score for that day, as described by Johnson et al. [28]. The mean of the 3 daily health status scores represented each mouse's infection process during 3 days postinfection. The blood in heart and brain of all tested mouse were picked using inoculation loops, and then plates of MacConkey agar were crossed by inoculation loops and cultured at 37°C overnight.

#### (iv) Rat neonatal meningitis model

The abilities to induce septicemia and enter the central nerves system (CNS) for APEC strains were assessed by 5 days old, specific-pathogen-free Sprague-Dawley rats, as previously described [28], [32]. And *E. coli* MG1655 and NMEC strain RS218 acted as negative and positive controls, respectively. Groups of 12 rat pups were intraperitoneally inoculated with approximately 200 CFU of bacteria suspensions (20 µl) [32]. At 24 h postinoculation, rats were subsequently euthanized, and 25 µl of blood and 10 µl of cerebrospinal fluid (CSF) from each survivor for infected rat pup were obtained for quantitative cultures. The blood and CSF were plated on MacConkey agar to measure the bacteria concentration in the blood and indicate meningitis, respectively.

## Results and Discussion

### Sequencing and overview of the complete genome of APEC strain IMT5155

The complete genome of APEC strain IMT5155 was determined by initial *de novo* assembly of two shotgun sequencing runs and one paired-end sequencing run (8-kb insert paired-end library) followed by PCR gap-filling. The raw shotgun reads and paired-end reads were assembled into 121 contigs which were further assembled into eight scaffolds. The N50 contig size was 177,509 bp. The largest scaffold size was 4,907,543 bp (containing 56 large contigs). The second largest scaffold size was 191,765 bp (containing 14 large contigs) indicating that our raw assembly was highly continuous and that might be sequence of *E. coli* large plasmids. Primer pairs were designed to amplify the gaps between contigs. The PCR products were directly sequenced using a Sanger sequencer ABI 3730. For the shotgun runs, one run generated 132,755 reads (∼53 Mb) and the other generated 108,804 reads (∼47 Mb). The average read length of both shotgun runs was approximately 400 bp. The paired-end run generated 90,792 reads (∼26 Mb) with an average read length of approximately 300 bp. Over 99% of the total reads were assembled, resulting in approximately 23-fold coverage of the genome of APEC strain IMT5155.

The complete genome of APEC strain IMT5155 comprises 5,126,057 bp, existing as a circular chromosome of 4,929,051 bp and two plasmids of 194,170 bp and 2,836 bp. Glimmer 3.02 annotated 4,804 CDSs covering 87.87% of IMT5155 chromosome. In addition, 27 pseudogenes and 30 small CDSs in intergenic regions were identified (File C in File S3). p1ColV_5155_ contained 270 Glimmer-predicted CDSs (File D in File S3), and 6 CDSs were identified in p2_5155_. Moreover, 88 tRNA genes, 19 rRNA genes, and 1 tmRNA gene were identified in the IMT5155 chromosome (File C in File S3). The GC content of the IMT5155 chromosome is approximately 50.65%, which is similar to other reported *E. coli* genomes. By contrast, the two plasmids have GC% contents of 49.84% (p1ColV_5155_) and 42.21% (p2_5155_). The large plasmid, p1ColV_5155_, was identified as a ColV plasmid, which was widespread in ExPEC pathotypes, particularly in APEC pathotype[47], [48]. Table A in File S2 summarizes the general genomic features of IMT5155 genome. Among 5,144 Glimmer-annotated CDSs found in IMT5155 genome, 5,053 (∼98.2%) could be matched to genes in the NCBI nr database (December, 2013).

### Whole-genome phylogenetic analysis of IMT5155 compared with other *E. coli* pathotypes

**Figure 1:**
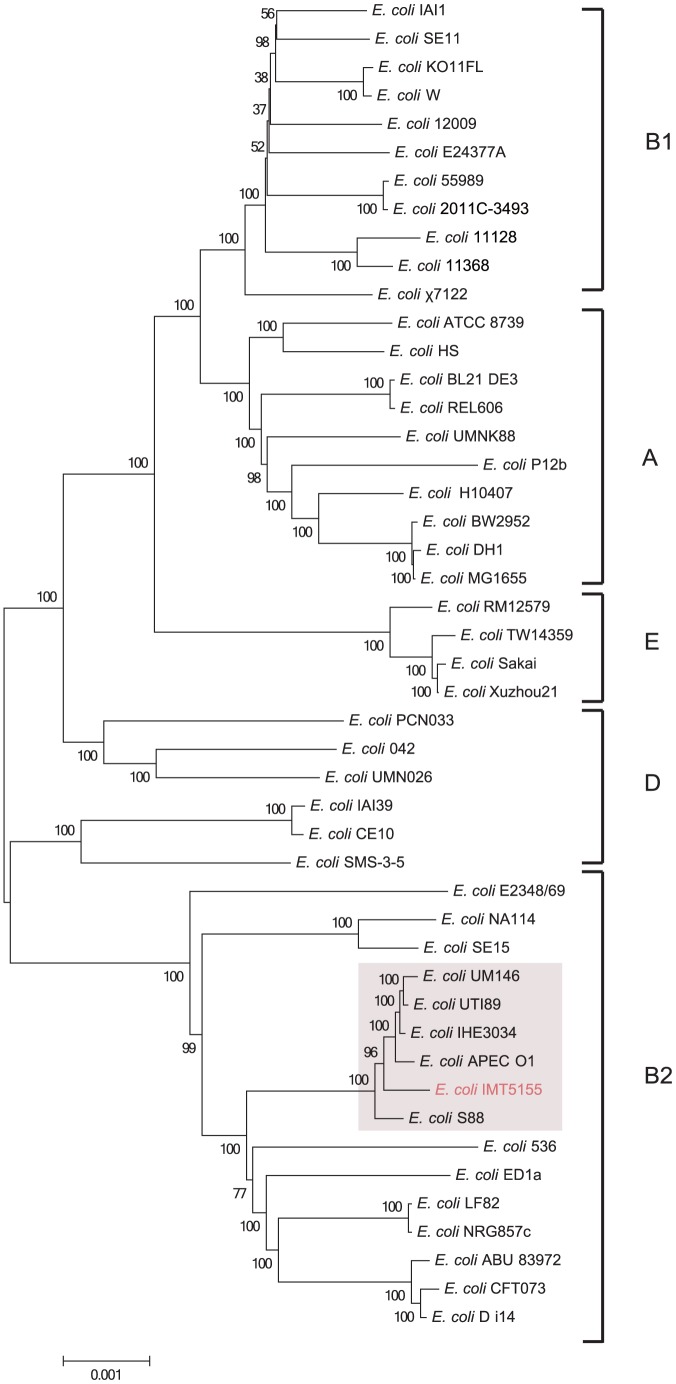
Phylogenomic tree (1,782 concatenated core genes, 1.61 Mb) of 47 *E. coli* strains. All MrBayes with the GTR+G+I substitution model (BMCMC) was used for the reconstruction of the phylogenomic tree. The chain length was set to 10,000,000 (1 sample/1000 generations). 47 *E. coli* strains clearly divided into monophyletically phylogroups (A, B1, B2, D, and E), and ST complex 95 strains were highlighted in phylogenomic tree. 47 *E. coli* genomes data was listed in File A in File S3.

Whole-genome-derived phylogeny of common genomes can accurately illustrate evolutionary relationships among different commensal and pathogenic *E. coli* variants [49]. The genomes of IMT5155 and another 46 *E. coli* strains were selected for mapping the whole-genome evolutionary phylogeny, ranging from a commensal K12 strain, through intestinal pathogenic strains, to the highlighted extraintestinal pathogenic strains (Figure 1). MrBayes was used to construct a BMCMC phylogenetic tree to define the evolutionary phylogeny of 47 whole genome sequenced *E. coli* strains, based on *E. coli* common genes. The common genes identified from IMT5155 and the others 46 *E. coli* genomes comprised 1,782 genes and covered approximately 1.61 Mb. The result of phylogeny showed that 47 *E. coli* strains could be clearly divided into six monophyletic groups, which was similar to the whole-genome-based phylogeny by both Rasko and McNally et al. [26], [50] (Figure 1). In the phylogenetic tree, APEC strains IMT5155 and APEC O1 were located in B2 ExPEC cluster (Figure 1), and an APEC O78 strain χ7122 was located in B1 clade (Figure 1). The phylogenomic tree showed that ST complex 95 APEC dominant O1:K1 and O2:K1 serotypes strains (APEC O1 and IMT5155) have the closest evolutionary relationships with human ExPEC dominant O18:K1 (ST95 complex) strains (UTI89 and IHE3034).

### Identification of virulence determinants and genomic islands in the IMT5155 genome

Many virulence-associated factors were identified in IMT5155 genome (Table B in File S2). Adhesins, invasins, and iron uptake systems were critical for APEC/ExPEC pathogenesis, which typically promote motility, achieve the capability of adhesion to and invasion of host tissues, and conduct iron uptake for survival [51]–[53]. The predicted adhesins of IMT5155 genome were listed in Table B in File S2. Six different chaperone-usher adhesion determinants were identified at IMT5155 genome, including *fim*, *yqi*, *yad*, *auf*, *yfc,* and *fml* operons. APEC strains shared common invasion genes with NMEC strains isolated from patients with neonatal meningitis [28], [51]. Several microbial invasion determinants, including *Ibe* proteins, *yijP*, *aslA*, K1 capsule, and *Hcp* family proteins (Table B in File S2) which contribute to invasion of brain microvascular endothelial cells (BMECs), were identified at both APEC and NMEC pathotypes [46], [54], [55]. IMT5155 possessed ferrous iron transporters *FeoABC* and *SitABCD* (Table B in File S2). Unlike widespread siderophore enterobactin, IMT5155 contained three ExPEC specific pathogen-related siderophores for salmochelin, aerobactin, and yersiniabactin, which took important roles in APEC virulence [52], [56] (Table B in File S2).

The distinct genomic islands (GIs) of pathogens that encode various virulence factors are called pathogenicity islands (PAIs), which have a significant difference in GC content compared with the core genome, and some PAIs are usually integrated into tRNA genes [57]. In this study, 20 GIs, ranging from 4 to 96-kb, were annotated on the IMT5155 chromosome via PAIDB and IslandViewer (Table C in File S2). 14 GIs contained several potential virulence factors, as predicted by PAIDB forecast and NCBI BLAST analysis, and these islands could be considered as confirmed or presumed PAIs. Moreover, 5 prophage islands (GI-5, -6, -13, -18, and -19) were identified at IMT5155 chromosome. Among the five prophages, it seemed that GI-13 was a P4 family phage and GI-18 was a P2 family member. The coexistence of these two phages (a satellite and helper phage pair) was quite reasonable [58]. It was also likely that the GI-18 phage could produce two types of tail fibers by DNA inversion like phage Mu and several other phages [59], [60]. The detailed and precise information for each GI had been elucidated and listed at Table C in File S2. We then focused on a novel APEC O2 PAI (GI-12) and two GIs (GI-8 and GI-22) coding Type VI secretion systems.

**Figure 2:**
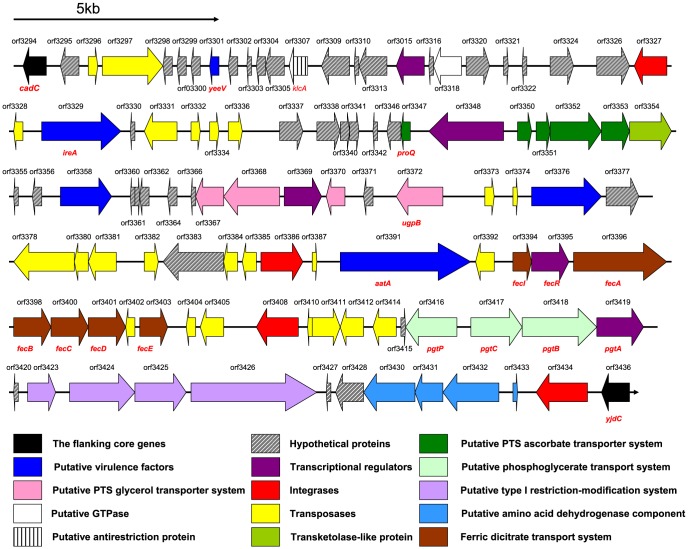
Chimeric feature and genetic context of PAI I_5155_ (GI-12). PAI I_5155_ was inserted between the *cadC* and *yidC* genes of *E. coli* core genome. Proteins encoded by the ORFs of PAI I_5155_ represented by arrows, and the direction of the arrows indicated the direction of transcription. The color keys for functions of these proteins were shown at the bottom.

A novel APEC O2 PAI (GI-12), termed PAI I_5155_, was identified from the IMT5155 chromosome, which inserted between the *cadC* and *yidC* genes of *E. coli* core genome, was adjacent to tRNA-Phe (Figure 2 and Table C in File S2). The total GC content of this island was 48.76%, below to the average GC content(50.65%)of IMT5155 chromosome. The size of PAI I_5155_ was approximately 94 kb, composed 105 ORFs. Proteins encoded by ORFs of PAI I_5155_ were shown in Figure 2 and Table C in File S2. PAI I_5155_ was absent in APEC O1 and other ExPEC genomes in this study, and only partial CDSs including several virulence/fitness factors (*aatA*, ireA, *fecIRABCDE*, and *pgtABCP*) were identified in pathogenicity islands of other *E. coli* pathotypes. For virulence factors encoded in PAI I_5155_, AatA of APEC autotransporter adhesin, IreA of iron-regulated virulence factor have been confirmed that they were involved in the pathogenicity of APEC/ExPEC [33], [61], [62], and other putative virulence genes need to be further identified (Figure 2 and Table C in File S2). Unlike other ExPEC, IMT5155 contained the ferric dicitrate transport system, which was previously reported to maintain *E. coli* growth under iron-limited circumstances and widespread among *E. coli* K-12, intestinal pathogenic *E. coli*, and *Shigella* strains [63]. For the putative metabolism/biosynthesis-related systems, those annotated genes of PAI I_5155_ were mainly distributed in ExPEC strains by BLASTN analysis. A putative transketolase-like protein, which was adjacent to a putative ascorbate-specific IIABC component of a PTS system, was also annotated in PAI I_5155_. In addition, like typical PAIs, PAI I_5155_ contained many mobility elements, including four integrases and multiple transposons, suggesting that horizontal gene transfer and genomic recombination were possibly involved in the evolution of PAI I_5155_ (Figure 2 and Table C in File S2). We identified a PAI I_5155_ analogue located in the chromosome of APEC strain DE205B (O2:K1), which was isolated in China (unpublished data) [45]. Therefore, PAI I_5155_ could be considered as a novel arrangement of these virulence factors and metabolism/biosynthesis-related systems. This island currently was only identified in APEC serotype O2 strains. Furthermore, roles of the putative virulence factors and metabolism/biosynthesis-related systems in pathogenicity and fitness of bacterial demands pending further research.

Type VI secretion systems (T6SSs) are distributed widely in many Gram-negative pathogenic bacteria [64]. IMT5155 carried two putative type VI secretion systems, which were located in GI-7 (32.2 kb) and GI-16 (28.2 kb) (Table C in File S2). GI-7, which was inserted between the *mltA* and *serA-*1 genes of B2 ExPEC core genome, was a region (GC content: 52.81%) adjacent to the tRNA-Met. GI-16 (GC content: 51.95%) located directly downstream of a tRNA-Asp, was inserted between the *yafT* and *ramA-*1 genes of *E. coli* core genome. GI-7 and GI-16 were respectively corresponding to T6SS1 and T6SS2, both of which have been recently described by Ma et al. [65]. The genes encoding secretion assembly components, including conserved core components of T6SS and additional unknown proteins [65], were located in GI-7 and GI-16 (Figure A in File S1). The typical T6SS1 (GI-7) was widely prevalent among the B2 and D ExPEC strains, and was elaborated to take roles in pathogenesis of APEC [28], [66]. However, it was reported that the T6SS2 was mainly encoded in virulent isolated of B2 ExPEC and might be a potential marker for B2 ExPEC, but not associated with ExPEC virulence [28], [65]. In order to identify whether T6SS2 can act as a potential marker for ExPEC dominant serotypes (O1, O2, and O18) strains, we detected almost all of the reported ExPEC O1:K1, O2:K1 and O18:K1 strains (genome sequences available online) and APEC isolates in our laboratory as previously described by Ma et al. [65] (Table D in File S2). We speculated that T6SS2 might be associated with ST95 ExPEC (serotypes O1, O2 and O18) strains, and those B2 phylogroup ExPEC (O1, O2, and O18) strains almost simultaneously contained two T6SSs (T6SS1 and T6SS2) (Table D in File S2).

### Comparative genomic analysis of IMT5155 with other *E. coli* pathotypes

**Figure 3:**
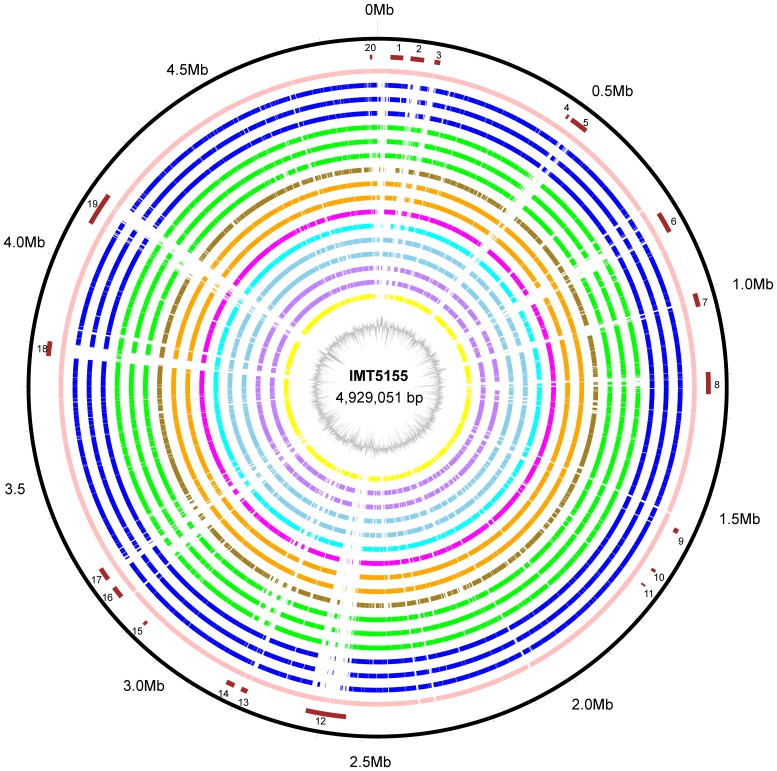
Comparative ORF analysis between IMT5155 and other *E. coli* strains. From outside to inside, the circles represent that: a) coordinate of IMT5155 genome; b) IMT5155 genomic island regions (red); c) IMT5155 (pink); d) APEC O1, IHE304, and UTI189 (blue); e) CFT073, ABU 83972 and NA114 (green); f) χ7122 (olive); g) UM146 and NRG857c (orange); h) SE15 (magenta); i) E2348/69 (cyan); j) CE10 and UMN026 (skyblue); k) O157 Sakai and O55:H7 RM12579 (purple); l) MG1655 (yellow); GC% of IMT5155 (calculated by 500 bp sliding window).

Comparative genomic analysis was performed using one by one alignment between IMT5155 genome and other 16 representative *E. coli* strains based on their evolutionary relationships and phenotypes. The general comparison of IMT5155 genome content with 16 *E. coli* strains was shown in Table A in File S2. The 16 representative strains encompassed typical commensal *E. coli*, highly pathogenic diarrhoeagenic *E. coli*, and extraintestinal *E. coli* strains. Four of these 16 *E. coli* strains were used as control references for comparative genomic analysis, including the commensal strains (MG1655 and SE15), EHEC strain O157 Sakai, and EPEC strain RM12579. IMT5155 shared different numbers of common chromosomal genes with these strains (Table E in File S2). The comparative chromosomal atlas of IMT5155 with those *E. coli* genomes is shown in Figure 3. The results showed that significant differences in genome content mainly focus on IMT5155 GIs regions (Figure 3). The distribution of IMT5155 GIs among these strains was shown in Table C in File S2. The commensal *E. coli* genomes were usually smaller than *E. coli* pathotypes, and harbored fewer genes, especially accessory genes i.e., genomic islands by genomic recombination than *E. coli* pathotypes [19], [49]. As described above, MG1655 harbored merely IMT5155 GIs homology loci (Figure 3 and Table C in File S2). Comparison between B2 phylogroup SE15 and IMT5155 reflected a similar result that only 4 IMT5155 GIs were present in SE15. The EHEC O157:H7 pathotype is a typical highly pathogenic diarrhoeagenic *E. coli* and highlighted the genomic plasticity for lateral gene transfer. EPEC strain RM12579 (O55:H7) is a precursor to O157:H7 pathotype [67], [68]. Both E phylogroup Sakai and RM12579 harbored merely IMT5155 GIs homology loci (Figure 3 and Table C in File S2), and Sakai shared the least numbers of chromosomal common genes with IMT5155 (Table E in File S2). The typical EPEC strain E2348/69 (serotype O127:H6) shares close evolutionary relationship with B2 ExPEC pathotypes, but has no common GIs with IMT5155. Two AIEC strains (UM146 and NRG857c) shared relatively largest numbers of common genes with IMT5155. UM146 and NRG857c had12 and 9 common GIs with IMT5155, respectively.

For 9 ExPEC strains in the comparative genomic analysis, APEC O1, IHE3034, and UTI89 exhibited closest phylogenetic relationship with IMT5155 (Figure 1). CFT073, ABU83972 and NA114 were in different subclades of phylogenetic tree relative to IMT5155, respectively (Figure 1). Our phylogenetic tree and previous studies revealed APEC ST23 serotype O78 strain χ7122 arose from distinct lineages with APEC O1 and IMT5155 [12]. In addition, CE10 and UMN026 belong to phylogroup D. The comparative genomic analysis showed that IMT5155 GIs, excepting for PAI I_5155_ and several prophage GIs, were highly conserved in APEC O1, IHE3034, and UTI89 (Figure 3 and Table C in File S2). Furthermore, IMT5155 shared the highest number of common chromosomal genes with IHE3034 (3,948; 83.0% of the total annotated CDSs in IHE3034 genome) (Table E in File S2). In contract, IMT5155 GIs were not widespread among CFT073, ABU83972, NA114, CE10, UMN026, and χ7122 (Table C in File S2). Moreover, 16 of the 20 genomic islands of IMT5155 were absent or poorly conserved in χ7122, and this result further reinforced the fact that ST23 APEC O78 strains lacked of conservation of virulence-associated genomic islands with ST95 APEC serotypes O1 and O2 strains (Figure 3 and Table C in File S2). Interestingly, the results showed that prophage GIs in IMT5155 exhibited partial or no homology among these ExPEC strains. These results showed that genomes of APEC O1 and IMT5155 shared significant genetic overlap/similarities with human ExPEC O18 strains UTI89 and IHE3034. Moreover, those GIs of IMT5155 that were widespread among APEC O1, IHE3034, and UTI89 might be involved in or contribute to the pathogenicity and niche adaptation of ExPEC O1/O2/O18 strains (phylogroup B2; ST complex 95).

### Sequence analysis and characterization of IMT5155 ColV plasmid p1ColV5155

#### (i) Analysis and characterization of the structure of p1ColV_5155_

**Figure 4:**
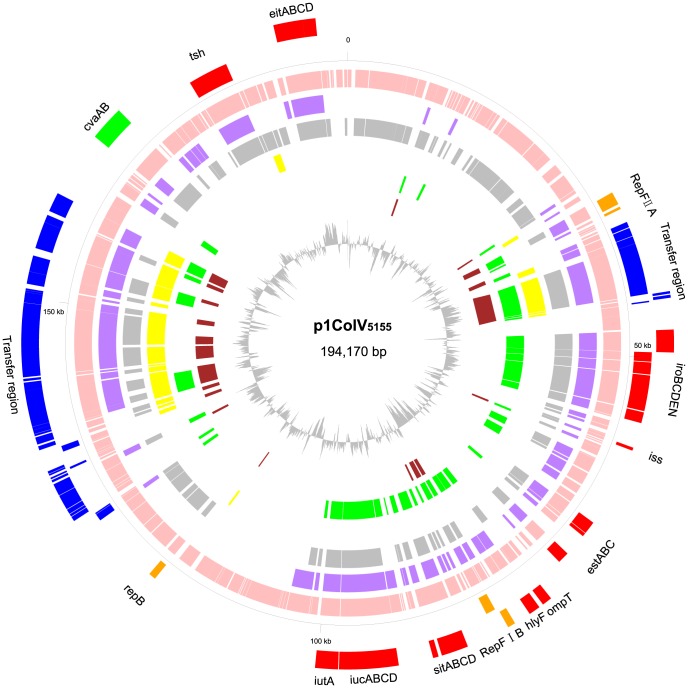
Comparative ORF analysis between p1ColV_5155_ and other plasmids. From inside to outside, the circles represent that: a) GC% (calculated by 500 bp sliding window); b) common ORFs in pUTI89 (brown); c) common ORFs in pO83_CORR (green); d) common ORFs in pMAR2 (yellow); e) common ORFs in pAPEC-O2-ColV (grey); f) common ORFs in pAPEC-O1-ColBM (purple); g) p1ColV_5155_ (pink); i) highlighted functional ORFs in the negative strand of p1ColV_5155_; j) highlighted functional ORFs in the positive strand of p1ColV_5155_ (orange: RepF IIA, RepF IB, repB; blue: Transfer regions; red: virulence related genes; green: cvaAB locus).

The IMT5155 strain harbored a 194-kb ColV plasmid, termed p1ColV_5155_, which have been described elsewhere [69]. p1ColV_5155_, which was depicted in a circular map (Figure 4), comprised 214 CDSs, encoding virulence-related proteins, plasmid conjugal transfer proteins, mobile genetic elements, and hypothetical proteins. The number and percentage of common genes of p1ColV_5155_ and the other *E. coli* pathotypes' plasmids were listed in Table F in File S2. p1ColV_5155_ shared more common genes with pAPEC-O2-ColV and pAPEC-O1-ColBM than the other large plasmids in other *E. coli* pathotypes (Table F in File S2). In an effort to better define p1ColV_5155_ backbone, classical circular genetic map was applied for comparative CDSs analysis of the p1ColV_5155_ with five other large plasmids (pAPEC-O2-ColV, pAPEC-O1-ColBM, pUTI89, pMAR2, and pO83-CoRR), three (pUTI89, pMAR2, and pO83-CoRR) of which acted as references for homology analysis (Figure 4). Plasmids pUTI89, pMAR2, and pO83-CoRR were respectively present in UTI89, E2348/69 and NRG 857C, which shared close evolutionary relationships with IMT5155 in the preceding section. In addition, synteny analysis between CDSs inp1ColV_5155_and the above five plasmids were also performed (Figure B in File S1). For the Tra genes region, we identified the detailed locations of p1ColV_5155_ homologous genes among those five plasmids. The common genes of p1ColV_5155_ with pAPEC-O2-ColV and pAPEC-O1-ColBM were mainly concentrated in virulence and plasmid conjugal transfer regions. The conjugative transfer system regions of pUTI89 and pMAR2 also shared high identity with that regions of p1ColV_5155_. However, the common genes between pO83-CoRR and p1ColV_5155_ were mainly located in the virulence region (Figure 4).

#### (ii)Virulence-associated genes of p1ColV_5155_

ColV plasmids are generally present in ExPEC strains and contain a series of virulence genes [70]. Several genes of ColV plasmids, identified as being involved in APEC virulence and defined the APEC pathotype [47], [48], [71], [72], were found at two virulence regikbons of p1ColV_5155_. The first virulence region with the size of 62.1 kb was from *iroBCDEN* of the salmochelin cluster to *iucABCD* and *iutA* of the aerobactin cluster (Figure 4). The second region was a 24.3-kb virulence gene region from *cvaA* and *cvaB* of the ColV operon to *eitABCD* of a putative iron transport system (Figure 4). In particular, the first virulence region of p1ColV_5155_ was nearly identical to the conserved portion of pAPEC-O2-ColV and pAPEC-O1-ColBM [47], [48]. The second virulence region of p1ColV_5155_ was homologous to the variable portion of pAPEC-O2-ColV and pAPEC-O1-ColBM, including *cvaAB*, *tsh*, and *eitABCD* [47], [48] (Figure 4). However, the virulence genes' locus in p1ColV_5155_ variable portion was completely inverted to that of pAPEC-O2-ColV (Figure 4 and Figure B in File S1). Further analysis of variable portion revealed that p1ColV_5155_ contained intact *cvaA* and *cvaB* genes for ColV export, but lacked the *cvaC* gene for ColV synthesis and the *cvi* gene for ColV immunity (Figure 4). Obviously, p1ColV_5155_ neither contained ColB and ColM operons, which were the namesake traits of ColBM plasmids [48] (Figure 4). Therefore, this plasmid named as ColBM plasmid can be excluded, due to the namesake traits of ColBM plasmids. Even though without encoding *cvaC* and *cvi*, p1ColV_5155_ was preferred to be classified as a ColV plasmid, which might lose the intact ColV operon during p1ColV_5155_ evolution. One speculation is that p1ColV_5155_ may be a novel type of ColV plasmid with rearrangements during its evolution. The pathogenic role of the two virulence regions of p1ColV_5155_ might be correspondent to pVM01 of APEC strain E3, which was highly similar to pAPEC-O2-ColV and pAPEC-O1–ColBM [47], [48], [72]. The conserved section of the pVM01 virulence region was clearly shown to be associated with the virulence of APEC strains. However, the variable sections of this plasmid were not directly associated with APEC virulence [72]. We speculated that the conserved section of p1ColV_5155_ virulence region might be involved in virulence of IMT5155.

#### (iii)Replication and transfer regions of p1ColV_5155_

Two replication regions were found in the chromosome of p1ColV_5155_: RepFIIA and RepFIB replicons (Figure 4). The first is a 33.4 kp region encompassing mostly predicted conjugal transfer genes of p1ColV_5155_, and the second is a 7.8 kp region contained another three conjugal transfer genes adjoining RepFIIA (Figure 4). The plasmid transfer region of p1ColV_5155_ was slightly different from that of pAPEC-O2-ColV, which contained a complete plasmid conjugal transfer region [47].

### The distribution of 10 sequenced B2 ExPEC pan-genome virulence genes among 46 sequenced *E. coli* strains

*E. coli* is highly evolved and adapted to the different specific environment. Recent findings show that the frequency of core genome recombination appears a striking decrease from intestinal commensal, through intestinal pathogenic strains, to phylogroup B2 ExPEC strains. Phylogroup B2 ExPEC strains are pathogenic variants, which show highly environmental adaptability with recombination being restricted [26], [73]. Comparative genomic analysis of IMT5155 with other *E. coli* pathotypes showed that APEC dominant O1 and O2 serotypes strains (phylogroup B2; ST complex 95) shared significant genetic overlap/similarities with human ExPEC dominant O18 strains (IHE3034, and UTI89), and could be distinguished from APEC O78 strain χ7122, commensal *E. coli*, and IPEC. Accordingly, B2 ExPEC strains should harbor typical ExPEC-specific virulence factors, which could endue ExPEC a selective advantage to adapt/colonize to extraintestinal specific niches during infection relative to intestinal pathogenic strains.

**Figure 5:**
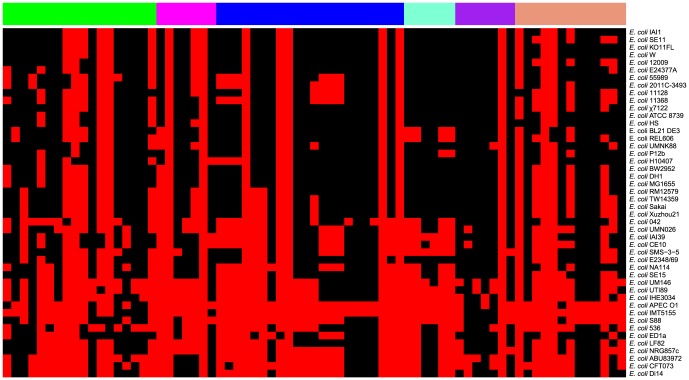
The distribution diagram of 10 sequenced B2 ExPEC pan-genome virulence genes among 46 *E. coli* strains. The uppermost row showed six classified clusters: 1, adhesins, green; 2, invasins, magenta; 3, iron acquisition/transport systems, blue; 4, polysialic acid synthesis, aquamarine; 5, toxins, purple; 6, others, darksalmon. Right side of the vertical line showed *E. coli* strains that were consistent with phylogenetic tree (Figure 1). The red and black body showed distribution of virulence genes among these strains. A red line meant that the virulence gene of interest was present at a particular strain, while a black line implied the gene was absent.

In order to understand the relationship between virulence factors and genetic landscape of B2 ExPEC pathotypes, the distribution of 10 sequenced B2 ExPEC pan-genome virulence genes among 46 sequenced *E. coli* strains was conducted to examine whether B2 ExPEC strains harbored typical ExPEC-specific virulence factors (i.e., determining whether there were significant differences for the distribution of B2 ExPEC virulence genes among different *E. coli* pathotypes) [51]. The pan-genome of sequenced 10 B2 ExPEC strains contained 10,399 orhthologous gene families. The VFDB database predicted 287 virulence genes among these orhthologous genes. 73 virulence-associated genes were manually confirmed among these 287 virulence genes and classified as six categories: adhesins, invasins, toxins, iron acquisition/transport systems, polysialic acid synthesis, and other virulence genes. The details of 73 virulence genes of 10 sequenced B2 ExPEC pan-genome and their distributions among 46 sequenced strains were shown in Figure 5 and Table B in File S2. The distribution diagram showed that 10 sequenced B2 ExPEC pan-genome virulence genes were significant occurring in extraintestinal pathogenic strains compared with commensal and diarrhoeagenic *E. coli*, and several virulence genes were only present among ExPEC strains, such as fimbrial adhesins (*yqi*, *auf*, and *papG*), invasins (*ibeA* and *Hcp*), almost of toxins, and others (Figure 5 and Table B in File S2). The distribution of 10 sequenced B2 ExPEC pan-genome virulence factors provided a meaningful information for ExPEC-specific virulence factors, including several adhesins, invasions, toxins, iron acquisition systems, and others (Figure 5 and Table B in File S2), which were conserved in ExPEC pathotypes and contributed to ExPEC to adapte/colonize extraintestinal specific niches during infection. Moreover, these specific virulence factors might also provide valuable targets for the vaccines design.

Certainly, there may be strain-to-strain variation of the distribution of virulence genes in any specific strains (Figure 5). For example, compared with other B2 ExPEC strains, IMT5155 does not have F1C, P, and S fimbariaes, which are involved in UPEC pathogenesis [53]. We wondered whether there were specific genes or virulence factors to define the APEC pathotype. For 10,399 orhthologous genes of 10 sequenced ExPEC pan-genome, 239 genes were identified in IMT5155 genome relative to the other 9 B2 ExPEC strains (Table G in File S2), and 202 genes were present only in APEC O1, and 24 genes were only common present in APEC strains (IMT5155 and APEC O1) compared with the other 8 B2 ExPEC strains (data not shown). The hypothetical genes and prophage genes were predominant among those specific genes for each APEC strains, and only five virulence genes (*aatA*, *eitA, eitB, eitC,* and *eitD*) were identified among 24 common genes. Moreover, 600 orhthologous genes were identify as NMEC-specific genes. Similarly, the majority of NMEC-specific genes were prophage genes and hypothetical genes, and no virulence factors were only present in NMEC (data not shown). Even though 3462 UPEC-specific genes among 10,399 orhthologous genes of 10 sequenced ExPEC pan-genome were identified in six UPEC strains, almost all virulence genes identified in UPEC strains were present among some APEC and UPEC strains. Therefore, there may be slight different distributions of virulence genes for an individual ExPEC strain, but no specific type of virulence genes to define B2 ExPEC subpathotype. The distribution analysis of 10 sequenced B2 ExPEC pan-genome virulence factors were further considered that phylogroup B2 APEC might not be differentiated from group B2 human ExPEC pathotypes (NMEC/UPEC), because two APEC O1 and O2 strains shared ExPEC-specific virulence factors with human ExPEC pathotypes. Furthermore, these results also support the previous findings that phylogroup B2 APEC isolates share remarkable similarities with human ExPEC pathotypes, and might pose a potential zoonosis threat [5], [9], [10], [27], [74].

### Virulence assessment of APEC O1:K1, O2:K1 and O78 serotypes isolates

The pathogenicity and zoonotic potential of APEC O1:K1 and O2:K1 serotypes isolates, including IMT5155 and several strains isolated in China, were assessed with four animal models [5], [28], [32], [45], [46]. In addition, one ST23 APEC O78 strain CVCC1553 and an APEC non-dominant serotype strain Jnd2 (B2; ST95; O39:K1) were also included in the virulence assessment. The strains APEC O1, NMEC RS218, and UPEC CFT073 were used as the positive control, while *E. coli* K-12 MG1655 and CVCC1531 were used as negative control [5], [28], [32], [45], [46]. The detail information of these 13 selected strains was shown in Table H in File S2.

The virulence of the selected APEC O1:K1, O2:K1, and O78 strains for natural reservoir were assessed by chicken embryo lethality assay (ELA) and chick colisepticemia model for avian colisepticemia. In ELA assay, the mortalities for un-inoculated, PBS-inoculated, Jnd2, and CVCC1531 inoculated embryos were not obviously different from the negative control MG1655, while seven APEC O1:K1, O2:K1, and O78 strains were significantly different from the negative control MG1655 (*P*<0.05) (Table 1). No significant differences existed among the seven APEC O1:K1, O2:K1, and O78 strains compared to the ELA-positive control strain APEC O1 (high pathogenicity) (Table 1). For chick colisepticemia assay, the mortalities, rates of reisolation from the chick organs, and lesion scores were evaluated. Similarly to ELA results, seven APEC O1:K1, O2:K1, and O78 strains were significantly different from the negative control MG1655 (*P*<0.05) (Table 2) (the original data shown in File E in File S3), while no significant differences were observed among the seven APEC O1:K1, O2:K1, and O78 strains compared to the high-pathogenicity control strain APEC O1 (Table 2). Therefore, based on the results of two models for avian colisepticemia, seven selected APEC O1:K1, O2:K1, and O78 strains was categorized as being highly virulent for natural reservoir. Recent reports show ExPEC isolates of same clonal group could be different for virulence genotypes, because acquisition of accessory virulence traits might be distinct evolutionary paths for strain-to-strain variation [8], [9], [32]. The virulence genotypes among APEC O1:K1 and O2:K1 strains showed slight differences (Table H in File S2), although the virulence for avian colisepticemia were similar (*P*≥0.05). Four APEC O2:K1 strains showed almost similar virulence genotypes, and *iucD* and *iroN* were absent in Fy26 and DE205B (Table H in File S2). For the virulence genotypes among three APEC O1:K1 strains, the two O1:K1 isolates (Jnd25 and CVCC249) in China did not harbor *ibeA* (GimA island) and *aatA* genes (APEC autotransporter adhesion) compared to APEC O1. The results of ELA assay and chick colisepticemia model showed that Jnd2 was a low-pathogenicity isolate compared to APEC O1 (*P*<0.05), even though previous studies claimed that ST95 B2 strains exhibited enhanced ExPEC virulence [8], [75]. There were significant differences between Jnd2 and APEC O1:K1/O2:K1 isolates that Jnd2 genomic did not harbor the typical T6SS1 (GI-7 for IMT5155), *vat*, and *ireA*, which are specifically required for survival and virulence during APEC infection [28], [62], [66], [76] (Table H in File S2). In short, combined pathogenicity tests with comparative genomic analysis, we confirmed that APEC O1:K1 and O2:K1 strains, including IMT5155 and several strains isolated in China, are extraintestinal pathogenic variants for high pathogenicity during infecting avian hosts, which is consistent with previous studies [5], [24], [26]–[29], [32].

**Table 1:** Mortality rates among chick embryos infected with APEC strains.

Strain	Mortality rate^d^	P value vs^a^:	
		MG1655	APEC O1
Uninoculated	0/10	1.0	<0.001
PBS	1/10	0.416	<0.001
MG1655^b^	3/20		<0.001
IMT5155	17/20	<0.001	0.306
Fy26	19/20	<0.001	0.179
DE164	17/20	<0.001	0.306
DE205B	18/20	<0.001	0.271
Jnd25	17/20	<0.001	0.306
CVCC249	16/20	<0.001	0.276
Jnd2	6/20	0.162	<0.001
CVCC1553	16/20	<0.001	0.276
CVCC1531	4/20	0.296	<0.001
APEC O1^c^	25/30	<0.001	

a *P* value measured by Fisher's exact test.

b Negative control for the ELA.

c Positive control for the ELA.

d Data mean the number of dead embryos/total number of embryos tested.

**Table 2:** Lethality in 1-day-old chicks for intratracheal inoculation with APEC isolates.

strain	Mortality rate*^cf^*	Reisolation rate (air sacs)*^df^*	Reisolation rate (blood) *^df^*	Reisolation rate (brain) *^df^*	Mean lesion score^e^
PBS	0/10^f^	1/10^f^	0/10^f^	0/10^f^	0.1 f
MG1655^a^	0/10	2/10	0/10	0/10	0.2
IMT5155	6/10	10/10	8/10	8/10	2.3
Fy26	6/10	10/10	9/10	7/10	2.5
DE164	5/10	9/10	9/10	7/10	2.3
DE205B	7/10	10/10	9/10	9/10	2.5
Jnd25	7/10	10/10	10/10	8/10	2.5
CVCC249	6/10	9/10	9/10	7/10	2.3
Jnd2	1/10^f^	5/10	3/10	0/10	0.9
CVCC1553	8/10	10/10	10/10	4/10	2.5
CVCC1531	0/10^f^	3/10^f^	0/10^f^	0/10^f^	0.3
APEC O1^b^	6/10	9/10	8/10	6/10	2.3

a Negative control for chick colisepticemia model.

b Positive control for chick colisepticemia model.

c Data mean the number of dead chicks/total number of chicks tested.

d Data mean the number of chicks from which the APEC strain was reisolated/total number of chicks tested.

e Mean of lesion scores (ranked from 0 to 3 due to occurrence of airsacculitis, pericarditis, and perihepatitis) for 10 chicks tested.

f Values are not significantly different (*P*≥0.05 by Fisher's exact test) with the negative control.

Previous reports put forward the hypothesis that APEC strains have zoonotic potential [6], [8], [9], and it is confirmed that a subset of APEC ST95 serotype O18 isolates could cause systemic disease in chickens and murine models of human ExPEC-caused septicemia and meningitis [32]. Our comparative genomic analysis further showed that IMT5155 shared significant genetic overlap/similarities with APEC O1 and human ExPEC O18 strains (IHE3034, and UTI89), and O1:K1/O2:K1 strains are common among APEC isolates but which also found among human NMEC and septicemic isolates [6], [9]. Certainly, APEC O1 is unable to cause bacteremia or meningitis in the neonatal rat model and keep host specificity by unknown mechanisms [28]. Here, we assessed the zoonotic potential of IMT5155 and the other O1:K1/O2:K1 isolates through two murine models of human ExPEC-caused septicemia and meningitis. For mouse sepsis assay, no mortalities were observed among mouse intraperitoneally inoculated (approximately 10^7^ CFU) with Jnd2, CVCC1531, APEC O1, CFT073, and MG1655 (Table 3) (the original data shown in File F in File S3). The data also showed that six APEC O1:K1/O2:K1 isolates (Jnd25, CVCC249, IMT5155, Fy26, DE164, and DE205B) and O78 strain CVCC1553 were not significantly different from the positive ExPEC reference strain RS218 (rate of mortality:100%)(*P*≥0.05) (Table 3), suggesting that those strains could have its ability to cause sepsis in the mouse through intraperitoneal inoculation. For rat neonatal meningitis assay, CVCC1531 and APEC strain jnd2 were unable to induce bacteremia in blood and CSF in neonatal rats (Table 4) (the original data shown in File G in File S3). The number of bacteria reisolated from the blood and CSF of rats infected with seven strains (Jnd25, CVCC249, IMT5155, Fy26, DE164, DE205B, and CVCC1553) were significantly higher than that of negative control (*P*<0.05) (Table 4). Moreover, IMT515 and five O1:K1/O2:K1 isolates in China showed comparable septicemia and meningitis in neonatal rats, since no significant differences in the blood and CSF counts were observed (*P*≥0.05). Our data demonstrated that IMT515 and five O1:K1/O2:K1 isolates were close to the high-level bacteremia in blood and CSF of RS218-inoculated neonatal rats, suggesting that these APEC O1:K1/O2:K1 isolates were able to cause septicemia and meningitis in neonatal rats. Like the subset of APEC ST95 serotype O18 isolates, our data confirmed that APEC O1:K1 and O2:K1 strains had zoonotic potential.

**Table 3:** Lethality of ICR mouse for intraperitoneal inoculation with APEC isolates and human ExPEC strains.

strain	Mortality rate*^cf^*	Reisolation rate (blood) *^df^*	Reisolation rate (brain) *^df^*	Mean lesion score^e^
PBS	0/10^f^	0/10^f^	0/10^f^	1.0 f
MG1655^a^	0/10	0/10	0/10	1.0
IMT5155	10/10	10/10	10/10	5.0
Fy26	10/10	10/10	10/10	4.9
DE164	10/10	10/10	10/10	4.9
DE205B	10/10	10/10	10/10	5.0
Jnd25	9/10	9/10	9/10	4.7
CVCC249	10/10	10/10	10/10	4.9
Jnd2	0/10^f^	4/10	0/10^f^	1.1 f
CVCC1553	10/10	10/10	7/10	5
CVCC1531	0/10^f^	0/10^f^	0/10^f^	1.0 f
APEC O1	0/10^f^	7/10	0/10^f^	1.5 f
RS218^b^	10/10	10/10	10/10	4.9
CFT073	0/10^f^	8/10	0/10^f^	1.5 f

a Negative control for mouse sepsis model.

b Positive control for mouse sepsis model.

c Data mean the number of dead mouse/total number of mouse tested.

d Data mean the number of mouse from which the APEC/ExPEC strain was reisolated/total number of mouse tested.

e Mean of lesion scores (1 = healthy, 2 = minimally ill, 3 = moderately ill, 4 = severely ill, 5 = dead) for 10 mouse tested.

f Values are not significantly different (*P*≥0.05 by Fisher's exact test) with the negative control.

**Table 4:** Pathogenicities of APEC isolates in the neonatal rat meningitis model.

Strain	Inoculum (log_10_ CFU per animal)	Mortality rate c	Reisolation rate from blood of survivors d	Mean log_10_ CFU ml^−1^ (blood) e	Reisolation rate from CSF of survivors d	Mean log_10_ CFU ml^−1^ (CSF) e
PBS	0	0/12 f	0/12 f	0	0/12	0
MG1655 a	2.36	0/12	0/12	0	0/12	0
IMT5155	2.33	1/12 f	10/11	3.54	10/11	4.02
Fy26	2.34	0/12 f	12/12	3.57	10/12	4.10
DE164	2.31	0/12 f	12/12	3.41	11/12	3.95
DE205B	2.25	1/12 f	10/11	3.51	10/11	4.18
Jnd25	2.35	1/12 f	10/11	3.64	10/11	4.3
CVCC249	2.32	0/12 f	12/12	3.50	12/12	4.16
Jnd2	2.32	0/12 f	2/12 f	3.17	0/12 f	0
CVCC1553 f	2.34	0/12 f	7/12	2.85	7/12	3.51
CVCC1531	2.31	0/12 f	0/12 f	0	0/12 f	0
APEC O1 a	2.34	0/12 f	0/12 f	0	0/12 f	0
RS218 b	2.34	3/12	9/9	3.82	9/9	>4.57

a Negative control for the neonatal rat meningitis model.

b Positive control for the neonatal rat meningitis model.

c Data mean the number of dead rats/total number of rats tested.

d Data mean the number of rats from which the APEC/ExPEC strain was reisolated/total number of rat survivors.

e Mean number of *E. coli* isolates recovered from the blood and CSF of the rat survivors.

f Values are not significantly different (*P*≥0.05 by Fisher's exact test) with the negative control.

A subset of APEC ST23 serotype O78 isolates could be acknowledged as APEC-specific pathogens, because APEC O78 strains were clearly differentiated from serotypes O1, O2, and O18 by MLST, phylogroup, and virulence genotypes [9]. The APEC O78 strain χ7122 was used as a classic infection strain of APEC pathogenicity to identify O78-specific virulence genotype [12]. Comparative genomic analysis of IMT5155 with χ7122 was consistent with the description by Dziva et al. that χ7122 were distinct from APEC O1 and IMT5155, and close to human ST23 serotype O78 human ETEC strain [12]. We compared the virulence and zoonotic potential of APEC O78 strain CVCC1553 with ST23 intestinal pathogenic strain CVCC1531. Like APEC O1:K1 and O2:K1 isolates, CVCC1553 was categorized as being highly virulent for natural reservoir, and CVCC1531 was avirulent in ELA and chick colisepticemia model (Table 1 and Table 2). Meanwhile, both CVCC1553 and χ7122 caused low pathogenicity in the neonatal meningitis mode compared to RS218 and APEC O1:K1/O2:K1 isolates (Table 4) [32]. As discussed by Dziva et al., χ7122 acquired a different virulence gene repertoire via variation in the accessory genome enabling success in avian species, including virulence-associated large plasmids [12]. The virulence genotype of CVCC1553 showed that it also contained the conserved regions of large virulence plasmids (Table H in File S2). Our investigation further confirmed that APEC O78 strains could act as avian host-specific extraintestinal pathogenic variant of ST23 lineage to adapt/colonize to extraintestinal specific niches and establish a specific infection by an intratracheal route in avian host.

## Conclusions

The study presented here enriches our knowledge of IMT5155 and complements the *E. coli* genome data of O2 serotype and ST140 (ST complex 95). Our phylogeny analyses confirmed that IMT5155 was closest evolutionary relationship with APEC O1 serotype and human ExPEC O18 serotype strains (APEC O1, IHE3034, and UTI89; ST complex 95), which all belonged to phylogroup B2 and ST complex 95. Comparison of IMT5155 genome with other *E. coli* strains facilitated the identification of APEC/ExPEC genetic characteristics. Our results of comparative genomics showed that APEC dominant O1 and O2 serotypes strains (APEC O1 and IMT5155) shared significant genetic overlap/similarities with human ExPEC dominant O18 strains (IHE3034, and UTI89). The unique PAI I_5155_ (GI-12) was identified and conserved in APEC O2 isolates, and GI-7 and GI-16 encoding two typical T6SSs might be useful markers for the identification of ExPEC dominant serotypes (O1, O2, and O18) strains. IMT5155 contained a ColV plasmid p1ColV_5155_, and virulence genes in p1ColV_5155_ also defined the APEC pathotype. The distribution of 10 sequenced B2 ExPEC pan-genome virulence factors among 47 sequenced *E. coli* provided a meaningful evidence for phylogroup B2 APEC/ExPEC-specific virulence factors, including several adhesins, invasins, toxins, iron acquisition systems, and others, which contributed to ExPEC to adapte/colonize extraintestinal specific niches during infection. The pathogenicity tests of IMT515 and other APEC O1:K1 and O2:K1 serotypes isolates in China through four animal models showed that they were high virulent for avian colisepticemia and able to cause septicemia and meningitis in neonatal rats, suggesting these APEC O1:K1 and O2:K1 isolates had zoonotic potential. Our comparative genomics studies and the pathogenicity tests will promote the investigation of APEC/ExPEC pathogenesis and zoonotic potential of APEC, and pave the way to development of strategies in their prevention and treatment.

## Supporting Information

File S1 Figure A. Gene clusters of T6SS1 (GI-7) and T6SS2 (GI-16) in IMT5155 chromosome. Genes encoding conserved domain proteins were represented by the bule colors. And white arrows indicate other unknown proteins, which were not identified as part of the conserved core described by Ma et al. [65]. The flanking core genes were indicated by the black arrows. A) IMT5155 T6SS1 (GI-7); B) IMT5155 T6SS2 (GI-16). Figure B. Synteny analysis based on common ORFs between p1ColV_5155_ and 5 plasmids (pAPEC-O1-ColBM, pAPEC-O2-ColV, pMAR2, pO83_CORR, and pUTI89). Grey ribbons are common ORFs in p1ColV5155 and pAPEC-O2-ColV; Pink ribbons are common ORFs in p1ColV_5155_ and pAPEC-O1-ColBM; Yellow ribbons are common ORFs in p1ColV5155 and pMAR2; Purple ribbons are common ORFs in p1ColV5155 and PO83-CORR; Green ribbons are common ORFs in p1ColV5155 and PUTI89. Red blocks are repA genes; Purple blocks are *repB* genes; Blue blocks are *Tra* genes.
(RAR)

File S2 Table A. General feature of IMT5155 genome and other *E. coli* strains. Table B. The virulence factors in B2 ExPEC pan-genome among 10 *E. coli* strains. Table C. The genomic islands of IMT5155. Table D. The information of 15 ExPEC isolates for simultaneous presence of T6SS1 and T6SS2. Table E. Common genes shared with IMT5155 for 15 *E. coli* strains. Table F. The number and percentage of common genes of other *E. coli* pathotype's plasmids shared with p1ColV_5155_. Table G. The specific genes of IMT5155 relative to other 9 B2 ExPEC strains. Table H. The detail information of the 13 selected strains for pathogenicity testing.
(RAR)

File S3 File A. Detailed description for 47 *E. coli* genomes data. File B. The scripts for comparative genomic analysis. File C. Detailed description for annotated ORFs in the chromosome sequence of IMT5155. File D. Detailed description for annotated ORFs in p1ColV_5155_. File E. The original data for chick colisepticemia assay. File F. The original data for mouse sepsis assay. File G. The original data for rat neonatal meningitis assay.
(RAR)
